# Ising Model Reprogramming of a Repeat Protein's Equilibrium Unfolding Pathway

**DOI:** 10.1016/j.jmb.2016.02.022

**Published:** 2016-05-08

**Authors:** C. Millership, J.J. Phillips, E.R.G. Main

**Affiliations:** School of Biological and Chemical Sciences, G.E. Fogg Building, Queen Mary, University of London, Mile End Road, London, E1 4NS, UK

**Keywords:** CTPRs, consensus tetratricopeptide repeat proteins, GuHCl, guanidine hydrochloride, 1-D, one-dimensional, protein design, protein folding, repeat protein, linear Ising model

## Abstract

Repeat proteins are formed from units of 20–40 aa that stack together into quasi one-dimensional non-globular structures. This modular repetitive construction means that, unlike globular proteins, a repeat protein's equilibrium folding and thus thermodynamic stability can be analysed using linear Ising models. Typically, homozipper Ising models have been used. These treat the repeat protein as a series of identical interacting subunits (the repeated motifs) that couple together to form the folded protein. However, they cannot describe subunits of differing stabilities.

Here we show that a more sophisticated heteropolymer Ising model can be constructed and fitted to two new helix deletion series of consensus tetratricopeptide repeat proteins (CTPRs). This analysis, showing an asymmetric spread of stability between helices within CTPR ensembles, coupled with the Ising model's predictive qualities was then used to guide reprogramming of the unfolding pathway of a variant CTPR protein. The designed behaviour was engineered by introducing destabilising mutations that increased the thermodynamic asymmetry within a CTPR ensemble. The asymmetry caused the terminal α-helix to thermodynamically uncouple from the rest of the protein and preferentially unfold. This produced a specific, highly populated stable intermediate with a putative dimerisation interface. As such it is the first step in designing repeat proteins with function regulated by a conformational switch.

## Highlights

• Ising model was constructed and fitted to helix deletion series of CTPR proteins.
• The analysis showed an asymmetric spread of stability between helices within CTPRs.
• Ising model's predictive qualities used to guide reprogramming through mutation.
• Introduced mutations increased thermodynamic asymmetry and uncoupled unfolding.
• Reprogrammed unfolding gives a specific intermediate with a putative dimer interface.

## Graphical Abstract

**Figure:**
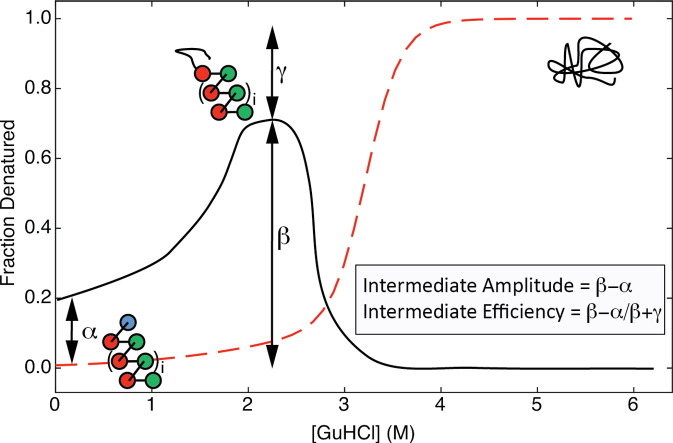

## Introduction

Repeat proteins are a diverse collection of superfamilies that are all composed of small modules (20–40 aa), which are stacked together to form stable non-globular domains [1], [2], [3]. Interestingly, the stacking of the repeated units results in modular structures that are dominated by regularised interactions from residues close in primary sequence (both inter- and intra-repeat). These distinctive features result in a quasi one-dimensional (1-D) structure that has made repeat proteins extremely attractive folds to engineer, design and use as protein folding/stability models (some examples include Refs. [3], [4], [5], [6], [7], [8], [9], [10], [11], [12], [13], [14], [15], [16], [17], [18], [19], [20], [21], [22]).

One elegant method used to dissect thermodynamic stability and folding cooperativity of both globular and repeat proteins is the fitting of their equilibrium denaturation curves or kinetics to Ising models. These statistical thermodynamic “nearest-neighbour” models have been used in many systems, both biological and non-biological, to describe order–disorder transitions [23]. Within the field of protein science, they have been used to probe helix to coil transitions, beta-hairpin formation, prediction of protein folding rates/thermodynamics and applied to interpret thermodynamic experiments that led to the postulation of downhill folding [23], [24], [25], [26], [27], [28], [29], [30], [31].

Importantly, the repetitive and quasi 1-D linear structure of repeat proteins, coupled with the ability to produce series of repeat deletion mutations, specifically lends their equilibrium thermodynamics and kinetics to be analysed with 1-D Ising models [16], [23], [32], [33], [34]. The most common Ising-based model used has been the 1-D homopolymer (also called a homozipper). Here, each arrayed element of a repeat protein is treated as an identical, equivalent independently folding unit. These interact with each other *via* nearest-neighbour pairwise interactions. Thus, folding is broken down into a linear series of identical interacting units. By globally fitting this model to chemical denaturations for a whole series of repeat proteins that differ only by their number of identical repeats, the intrinsic energy of a repeated unit and the interaction energy between the folded units can be delineated. Interestingly, once a subset of repeat proteins within a series has been fitted, the model can then be used to successfully predict the behaviour of all additional proteins within that series [16], [23].

The studies described above coupled with those on folding kinetics have highlighted how the modular structures of repeat proteins inherently influence their solution characteristics. In particular it has been shown that (i) the kinetic/equilibrium folding of repeat proteins are prone to the population of partially folded intermediate states and (ii) the population of such intermediates is determined by a subtle interplay between three factors—the total stability of the protein, the intrinsic stability of individual repeated units and the generation of favourable interfaces between the repeated units. More fundamentally, however, tuning the thermodynamic stability/folding pathways of repeat proteins may also provide a path for further exploitation. For example, the dominant kinetic folding pathway and/or the multistate folding of a repeat protein can be altered by introducing mutations that change either the inherent stability or inter-repeat packing of individual repeats within an ensemble [13], [14], [33], [35], [36]. Importantly, these studies open up the potential of exploiting the folding of repeat proteins in the form of a switch, that is, the engineered unfolding of part of a repeat protein to leave a folded intermediate unit with a compatible oligomerisation interface.

**Fig. 1:**
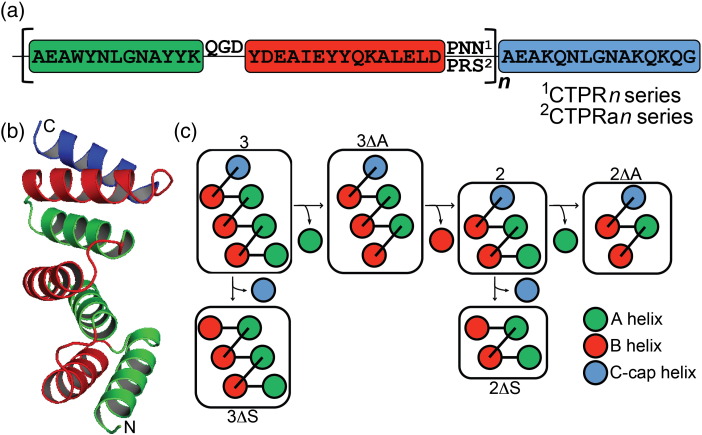
(a) The sequence of a CTPR repeat. It is made up of two α-helices (a and b coloured green and red, respectively) separated by a short loop. This sequence is repeated *n* number of times before there is a C-terminal capping helix (C-cap, coloured blue). The C-cap has also been called the “solvating helix” (S-helix) in previous studies [9], [15], [17], [33]. There have been two CTPR protein series constructed: CTPRn and CTPRan. The sequences of both are the same, except that the last two residues in each repeat (positions 33 and 34) are NN in the CTPRn series and RS in the CTPRan series. (b) Crystal structure of CTPR3. The α-helices are coloured according to their sequence (a, green; b, red; and c, blue). (c) Schematic showing the relationship and nomenclature between the mutants. Mutating out the N-cap terminal A-helix produces ∆A. Mutating out the C-cap terminal S-helix produces ∆S.

Here we investigate whether the predictive qualities of an Ising model can be used to reprogram the equilibrium folding pathway of a repeat protein. In particular we wanted to engineer a repeat protein to specifically unfold leaving a partially folded intermediate. This would be the first step toward introducing a folding-controlled switch-like function into repeat proteins. The system we chose to reprogram was our preferred system—designed consensus tetratricopeptide repeat proteins (CTPRs; Fig. 1). To achieve this, two new series of CTPR single-helix deletion mutants of differing intrinsic stabilities were constructed and their equilibrium unfolding was analysed with a more complex heteropolymer Ising model. The new deletion series coupled with the new Ising model analysis enabled a more detailed delineation of thermodynamic stability across CTPR ensembles. Specifically, it showed that the inherent structural asymmetry of the helices manifested in an asymmetric spread of thermodynamic stability, with the C-terminal helix (C-cap) less stable than the N-terminus (N-cap). The predictive nature of the fitted Ising model was then used to probe if the inherent asymmetry in stability could be exacerbated to uncouple folding and produce the desired specific, highly populated partially folded intermediate. After designing such a CTPR switch mutant, the protein was shown to biophysically behave as predicted—specifically unfolding at low concentrations of denaturant to leave a partially folded intermediate with the desired regions remaining structured.

## Results

We and the Regan laboratory have shown that designed consensus TPR proteins of sufficient overall stability tend to unfold *via* stable intermediates that have unfolded terminal helices [15], [33], [37], [38]. Moreover, removing the C-terminal α-helix from CTPR3 produces an interface compatible with oligomerisation that we have shown can be forced to associate through incubation with crosslinkers and through intein mediated ligation (Fig. S1; [39], [40]). Therefore, it is logical to reprogramme a CTPR protein to populate a specific intermediate that preferentially unfolds only one of its termini. To achieve this, the contribution to overall stability of the differing helices within wild-type CTPR proteins needs to be dissected in greater detail than in earlier studies.

### Global fit of CTPR unfolding to a heteropolymer Ising model

**Fig. 2:**
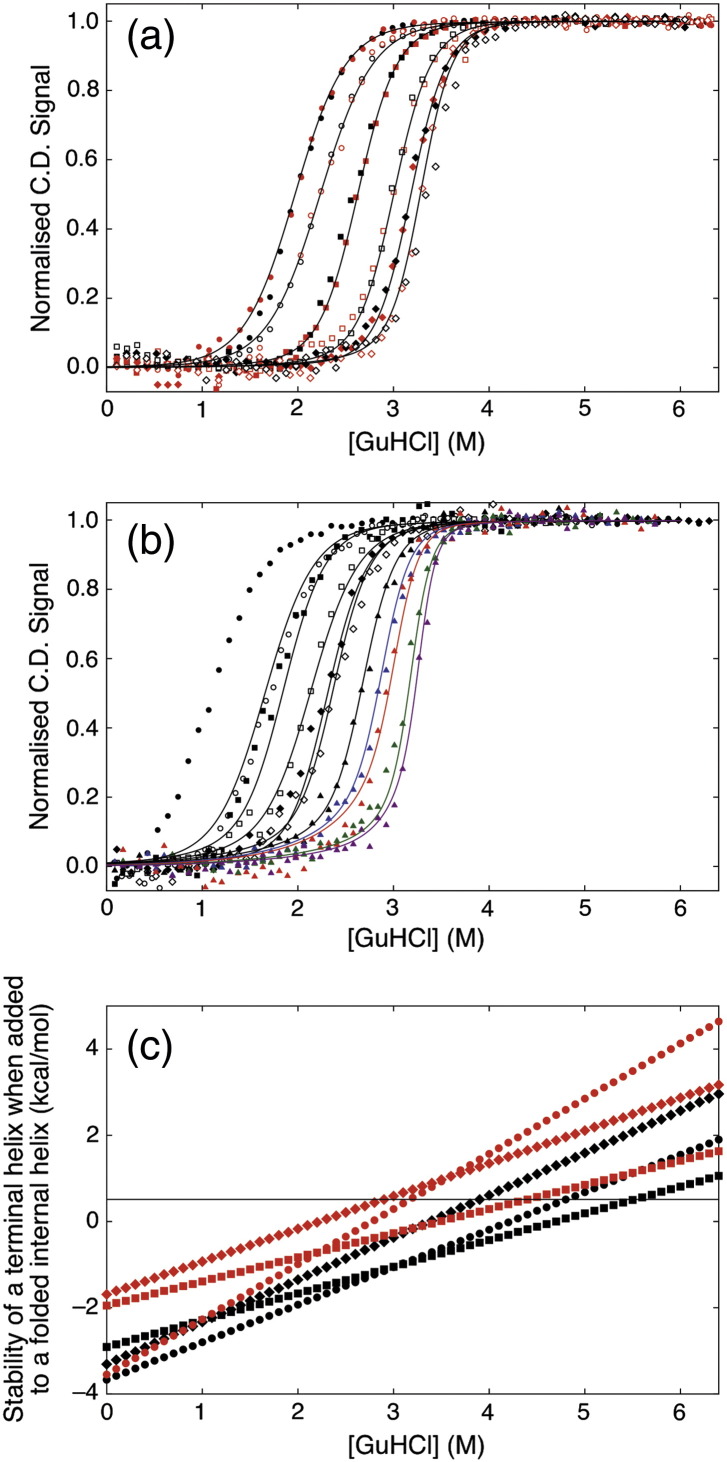
(a and b) GuHCl-induced equilibrium unfolding experiments of the CTPRn (a) and CTPRa (b) proteins in 50 mM phosphate (pH 7) at 10 °C shown as normalised CD signal. The fits correspond to the global fitting of each CTPR series to a heteropolymer Ising model. In images a and b: CTPR2 ∆ A (●,), CTPR2 ∆ S (○,), CTPR2 (■, ), CTPR3 ∆ A (,), CTPR3 ∆ S (◆,) and CTPR3 (◇,). CTPRa2 ∆ A (●), CTPRa2 ∆ S (○), CTPRa3 ∆ A () and CTPRa3 ∆ S (◆). The following data were obtained from published data in Main [9]: CTPRa2 (■), CTPRa3 (◇), CTPRa4 (▲), CTPRa5 (), CTPRa6 (), CTPRa8 () and CTPRa10 (). (c) Stabilities of terminal helices added to a folded CTPR ensemble as a function of GuHCl. An N-cap helix (■,), I helix (◆,) and a C-cap helix (●,). The CTPRn and CTPRa series are black and red, respectively.

**Fig. 3:**
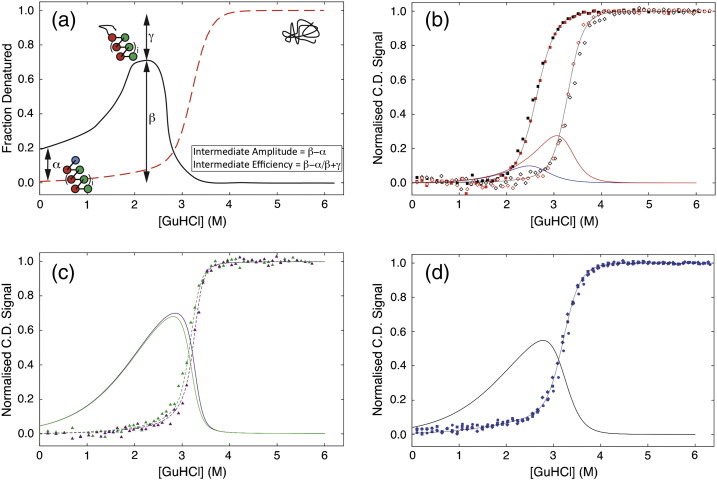
(a) Schematic of an engineered CTPR protein designed to unfold into a specific intermediate. The plot shows the equilibrium denaturation of a CTPR protein (dashed red line) overlaid with the fractional population of the C-cap unfolded CTPR intermediate at differing GuHCl concentrations (black line). This state can be characterised by its amplitude (*β* − *α*), its efficiency ([*β* − *α*]/[*β* + *γ*) and the degree to which the protein is fully folded at 0 M denaturant (*α*). A CTPR3 protein is schematically shown for effect. At 0 M GuHCl, it is fully folded; at 2 M GuHCl, the highest C-cap unfolded intermediate population is reached; and above 3.5 M GuHCl, it is denatured. (B and C) Ising model obtained fractional population of intermediates that only have a denatured C-cap helix for (b) CTPR2 (solid blue line) and CTPR3 (solid red line) and (c) CTPRa8 (solid green line) and CTPRa10 (solid purple line). The corresponding CTPR protein chemical denaturations with Ising fit (dashed lines) are shown for comparison. (d) Comparison of the GuHCl denaturations of CTPR3sw (,  and ) with the simulated CTPR3sw denaturation curve obtained from the Ising model (black dashed line) and the fractional population of CTPR3sw intermediates that only have an denatured C-cap helix (solid line).

Previously, we and the Regan laboratory used a 1-D homozipper Ising model to analyse equilibrium denaturation data from CTPR protein series that differed in size by whole TPR motifs [9], [16], [33]. However, such a system—the deletion series coupled with a homozipper model that treats all repeated elements in the protein as equal—cannot describe repeated units with differing stabilities (thermodynamic asymmetry). Therefore, we constructed eight new terminal helix deletion constructs on two series of CTPR proteins of differing intrinsic stabilities and performed guanidine hydrochloride (GuHCl)-induced chemical denaturations on each (CTPRn and CTPRan; Fig. 1a). Each series of chemical denaturations was then globally fit to a heteropolymer Ising model (Materials and Methods; Fig. 2). By using this system, the contributions to the overall stability from the differing helices can be differentiated and any differences in their intrinsic stability and interface energies delineated (Materials and Methods [23]). Moreover, the model permits both the calculation and simulation of populations of any specific topological state throughout any protein's denaturation curve. Thus, the behaviour of any putative partially folded intermediate populated during a proteins' chemical equilibrium unfolding can be interrogated and predictively modelled. Here, this was used to examine the population of any CTPR construct that was natively structured yet has a denatured terminal helix, at any given GuHCl concentration (Fig. 3).

The new deletion constructs consisted of CTPRn and CTPRan proteins that lacked either the N-terminal A-helix (N-cap), termed ∆A constructs, or the C-terminal S-helix (C-cap), termed ∆S constructs (where *n* = number of TPR repeat units; Fig. 1c). Both series were investigated as they differ by a destabilising double mutation (CTPRn: P**NN** to CTPRa: P**RS**) at the end of each TPR motif (Fig. 1a). All constructs were monomeric and folded with high α-helicity (Fig. S2). Chemical denaturations were performed using GuHCl at 10 °C in 50 mM phosphate (pH 7.0) and the structural transitions followed by monitoring changes in far-UV CD (ellipticity at 222 nm; Fig. 2a and b; Fig. S3). All of the constructs underwent single reversible unfolding transitions as the increase in GuHCl caused unfolding of their native structure to yield a fully denatured polypeptide. The Ising model used here was constructed of a linear algebraic series of equilibrium constants that represent an intrinsic folding stability term for each helix and an interfacial energy term between helices in a nearest-neighbour array (∆*G*_*i*_ and ∆*G*_*i* − 1 , *i*_, respectively). However, our newer model fitted nine variables that separated out the total stability of each CTPR ensemble into the stability and interface energies of the N-cap helix, internal I-helices and the C-cap helix (Materials and Methods).

A numerical solution of the Ising model was determined by globally fitting the chemical denaturation of each CTPR series. This consisted of fitting 12 denaturation curves of the CTPRn series (six proteins with repeated equilibrium denaturations) and 10 denaturation curves of the CTPRan series (CTPRa2∆A removed due to lack of native baseline). A single global minimum was ensured by seeding 1000 random searches, each with 1000 trajectories, using the Mathoptimizer module of Mathematica (Wolfram) (Fig. 2a and b). Our model fit well to the experimental data with a root mean square of residuals between the fit and the individual normalised equilibrium curves of < 3.5% of the data amplitude for the CTPR curves and between < 2% and < 5% for the CTPRan curves. Table 1 lists the values obtained for the fitted parameters.

### Determining the relative stability of individual α-helices

The stability of any CTPR ensemble, or part thereof, (*∆G*_0 → 1_^H_2_O^) can be calculated by simply summing energy terms obtained from the fitted variables (Table 1 and Table S3). In this manner, the differing energetic contributions of N-cap helix, C-cap helix and the internal I-helices can be easily calculated for each CTPR series (Table 1). These show that the individual helices of each CTPR/CTPRa protein do not have identical stabilities as described in previous homozipper analyses. Instead, the removal of the N-cap helix is more destabilising than removing an internal helix or C-cap helix. This is in agreement with a qualitative comparison of the chemical denaturation midpoints (Fig. 2, S.I. Fig. S3; Table S1). Although the calculated differences between helix stabilities are not large in magnitude at 0 M GuHCl (i.e., in water with no denaturant), they become more pronounced with the addition of GuHCl due to their differing denaturant dependence, represented in the Ising model as an *m*-value (Fig. 2c). Thus, both series of CTPR proteins possess some thermodynamic stability asymmetry. Interestingly, the stability asymmetry mirrors the structural asymmetry of each ensemble. Crystal structures of CTPR2 and CTPR3 show that a combination of different tertiary packing and primary sequences causes the C-terminal helix to be 25% more solvent exposed than the next most solvent exposed helix and more highly charged (AEA**KQ**NLGNA**KQ**KQG, calculated p*I* of 9.7 versus AEA**WY**NLGNA**YY**KQG, calculated p*I* 6.1 [17], [41]). The differing stabilities of the N-cap and C-cap helices also explains why crystal structures of certain CTPRs show the C-cap helix being preferentially unfolded (missing density) and its position occupied by the N-cap helix from another CTPR protein [42].

### Routes to reprogramming the equilibrium unfolding of a CTPR protein

The lower stability and higher denaturant sensitivity of the C-cap helix should cause it to preferentially unfold before the rest of the protein and notably before the N-cap helix (Fig. 2c). If accurate, it would suggest that engineering one terminal helix/repeat to possess a significant differential in stability to those in the rest of the protein would be a rational and logical route to reprogramming unfolding. To model if the less stable C-cap helix does bias the unfolding of the wild-type CTPR/CTPRa proteins, subpartition functions were defined representing those partially folded intermediates in the Ising model. Values determined from the global fitting to experimental data were then used to calculate fractional populations of intermediates states and to examine their sensitivity to denaturant while part of a known multi-state folding CTPR/CTPRa protein (Materials and Methods). Fig. 3a and b shows the plots for fractional protein population that possesses an intermediate with an unfolded C-cap helix, overlaid with the equilibrium denaturations for multistate-CTPR folding proteins: CTPR2, CTPR3, CTPRa8 and CTPRa10. Only these are shown as, under our conditions, the smaller CTPRa proteins appear to fold in a two-state manner [33]. Interestingly, all of the protein simulations do suggest that the slight differences in helical stability and denaturant dependence produce some of the desired unfolding bias; that is, populations of specific C-cap unfolded intermediate exist. These populations increase from close to zero in 0 M GuHCl (fully folded protein) to a maximum at intermediate concentrations of GuHCl before decreasing to zero again at higher concentrations of GuHCl (as further helices denature). For the CTPR2 and CTPR3 proteins, the model predicts only a small population of C-cap unfolded intermediate (10% and 27%, respectively). In contrast, CTPRa8 and CTPRa10 exhibit larger predicted populations of C-cap unfolded intermediate of 68% and 70%, respectively. The increase in population of the desired intermediate is therefore directly related to both a less stable and more denaturant sensitive C-cap helix and, importantly, an increase in ensemble stability. As, for example, although CTPR3 exhibits a high overall stability similar to CTPRa8/10, the N- and C-cap helices are too close in stability and denaturant sensitivity to exhibit any specific bias in intermediates populated while unfolding.

### Ising-led re-design

The Ising model simulations predict that engineering a CTPR/CTPRa protein to unfold *via* one specific highly population intermediate requires a high overall total protein stability and a suitable free energy differential/denaturant sensitivity between the C-cap and the other helices (Fig. 3). To test this hypothesis and test the Ising model's predictive qualities, it was logical to design and model a chimera that combined the stable core of the smaller CTPR3 from CTPRn series with the less stable C-cap helix of the CTPRa series (i.e., inserting the PNN to PRS mutation only between the third TPR motif and the C-cap helix; Fig. 1 [33]). The new construct was termed CTPR3sw (**CTPR3 sw**itch) and its chemical denaturation and C-cap unfolded intermediate function were simulated using the Ising model. Parameters were set using the experimentally fitted values for the N-cap (A) and internal (I) helices of CTPRn series and C-cap helix (S) from the CTPRan series (Fig. 3d). This predicted the protein to have a maximum C-cap unfolded intermediate of ≈ 55% (double that of the precursor CTPR3 protein) and further helical unfolding of only ≈ 15% at 2.8 M GuHCl. Thus, the CTPR3sw construct should have an intermediate that is populated to a similar extent as the larger CTPRa proteins and, crucially, can be experimentally validated due to its smaller size and lower number of identical repeats.

### Does CTPR3sw unfold as predicted?

#### Chemical Denaturation

**Fig. 4:**
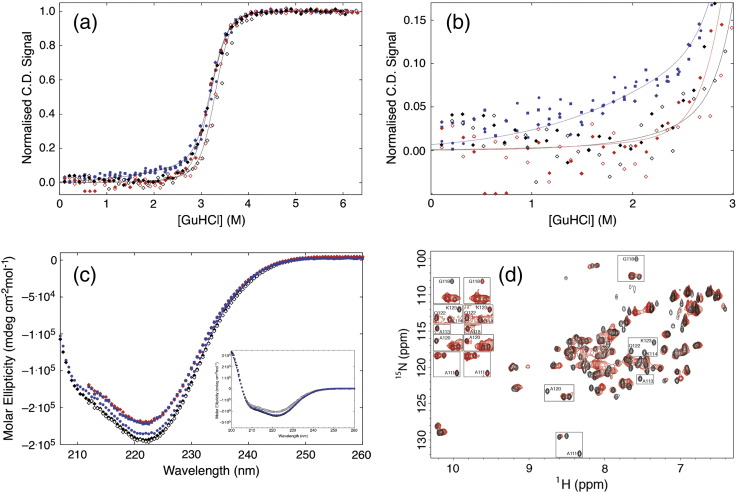
(a and b) Comparison of GuHCl denaturations of CTPR3sw (,  and ), CTPR3 ∆ S (◆,) and CTPR3 (◇,) in 50 mM phosphate (pH 7) at 10 °C. The small dashed black lines are the Ising model fits for CTPR3 ∆ S and CTPR3 from the global analysis of the CTPRn series, and the small dashed blue line is the Ising model's simulated denaturation curve for CTPR3sw. (b) is an enlargement of the native baseline of panel A. (c) Far UV CD wavelength scans of CTPR3, CTPR3∆S and CTPR3sw at 0 M GuHCl and 2 M GuHCl. In 0 M GuHCl, CTPR3sw () has the same ellipticity as CTPR3 (◆). However, in 2 M GuHCl, CTPR3sw () has the same ellipticity as CTPR3∆S () and not CTPR3 (◇). The inset figure shows the same Far UV CD wavelength scans of CTPR3, CTPR3 ∆ S and CTPR3sw at 0 M GuHCl extended to 200 nm. (d) HSQC spectra of CTPR3 (black) overlaid with CTPR3sw (red) at 0 M GuHCl. The resonances of the CTPR3sw C-cap amides with significantly reduced peak intensities are highlighted and shown at higher contrasts (insets).

To establish whether CTPR3sw specifically unfolds as predicted, the construct was expressed and purified. An initial characterisation showed the protein to be monomeric and possess the same α-helical structure as CTPR3 (Fig. S6A and B). As with all other CTPR constructs, its equilibrium chemical denaturation was marked by one major reversible unfolding transition with increasing GuHCl (Figs. 3d and 4). Excitingly, when the Ising model prediction was overlaid with the experimental data, there was extremely good agreement (Figs. 3d and 4). Thus, as predicted, CTPR3sw is less stable than CTPR3 (lower midpoint of denaturation), but with a midpoint that is the same as that seen for CTPR3 ∆ S. Significantly, there is an observable change in the slope of CTPR3sw's native baseline in comparison to any of the other CTPR variants (Fig. 4a and b): CTPR3sw's baseline has a positive gradient toward the first transition, whereas all other CTPR variants have native baselines that are essentially flat or possess negative gradient. Skewed pre-transition baselines, such as observed here for of CTPR3sw, are essentially identical to the skewed pre-transitions seen in a number of highly studied marginally cooperative folding domains [43], [44], [45], [46]. In each case, the skewed transition reflects partial unfolding and thus a thermodynamic uncoupling of folding elements. Triplicate repeated denaturations of CTPR3sw confirmed that the baseline changes observed were reproducible and significant (Fig. 4a and b). When the ellipticity was corrected for concentration, the maximum difference in baselines between the two proteins occurs from 2 M GuHCl and equates to ≈ 10% of the signal at 222 nm (Fig. 4a and b).

The baseline change was confirmed by recording CD wavelength scans for CTPR3sw, CTPR3 and CTPR3 ∆ S, with and without 2 M GuHCl (Fig. 4c). These show that in 0 M GuHCl, the ellipticity of CTPR3sw is the same as CTPR3; that is, they have the same helical content. However, at 2 M GuHCl, the ellipticity of CTPR3sw has reduced to the same as that seen for CTPR3 ∆ S. In comparison, CTPR3 remains relatively unperturbed in 2 M GuHCl. Thus, at or above this concentration, CTPR3sw seems to have the same α-helical content as CTPR3 ∆ S and undergoes a denaturation with the same midpoint as CTPR3 ∆ S. We interpret this to mean that CTPR3sw is preferentially unfolding helical structure from a seven-helix CTPR3-like state to a six-helix CTPR3 ∆ S-like state prior to the main denaturation transition.

#### ^15^N HSQC NMR

To ascertain whether the change in α-helical CD signal observed at low GuHCl concentrations was due to the unfolding of CTPR3sw's C-cap helix, heteronuclear single quantum coherences (HSQCs) of ^15^N-labelled CTPR3 and CTPR3sw in 0 M GuHCl and under low GuHCl molarities were recorded and compared (CTPR3—assignment as previously published [15]; CTPR3sw—Materials and Methods). There is, as expected, peak overlap in the spectra due to the near-identical sequence and structure of the TPR modules. However, sufficiently dispersed amide cross-peaks are present throughout both the CTPR3 and CTPR3sw spectra to report on the dynamics of each α-helix. For example, there are seven assigned amide peaks from the 15 residues in the C-cap helix of CTPR3sw (Fig. 4d).

#### HSQCs in 0 M GuHCl

Comparison of the HSQC at 0 M GuHCl of CTPR3 with that of CTPR3sw shows similarly dispersed spectra, with little perturbation in chemical shifts between corresponding CTPR3/CTPR3sw cross-peak amide protons (Fig. 4d). However, there is a specific and distinct reduction in the amide peak intensities for residues within CTPR3sw's C-cap helix (Fig. 4d). The loss of intensity can be caused either by peak broadening due to exchange or by conformational fluctuations (e.g., local unfolding). This suggests that the native state of CTPR3sw has a C-cap helix that is more structurally dynamic than that of CTPR3.

#### HSQCs in low [GuHCl]

**Fig. 5:**
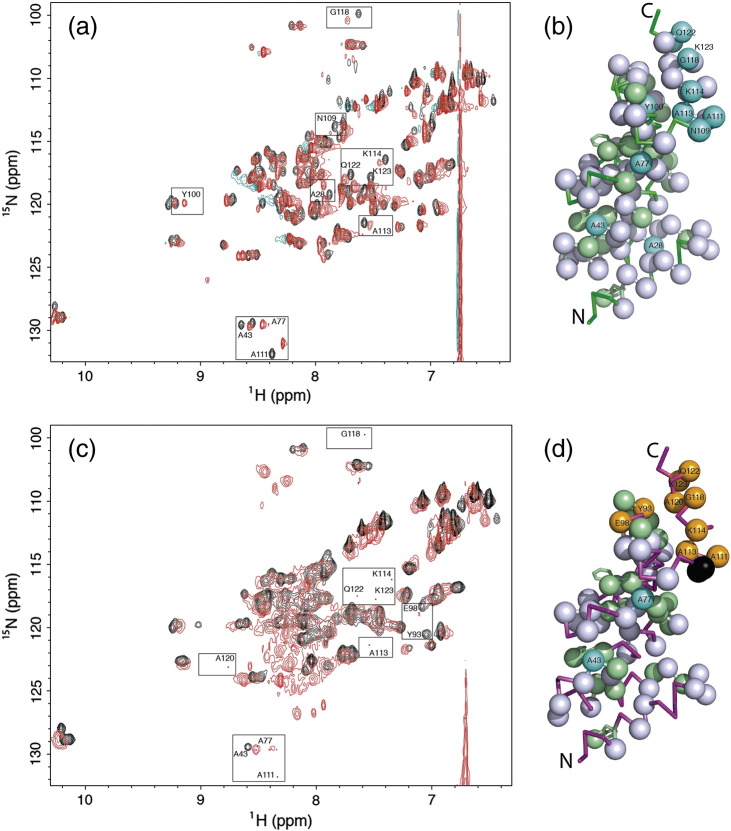
Overlaid HSQC spectra for CTPR3 (a) and CTPR3sw (c) obtained at 0 M GuHCl (black) and on addition of 1.6 M GuHCl (red). The resonances of shifted amides in either CTPR3 or CTPR3sw are highlighted. Images b and d map the non-overlapping amide peaks movements from panels a and c onto the CTPR3 crystal structure, respectively. The Cα's of assigned amides or side-chain indole NHε1 of the Trp residues are represented as spheres. Those that remain unchanged when 1.6 M GuHCl was added are coloured white (∆δ ≤ 0.04), those that move with a chemical shift perturbation (∆δ) of 0.04 to 0.22 are coloured from pale blue to cyan (respectively) and those that moved into a crowded region and therefore cannot be assigned with certainty are displayed in orange. Assigned residues that are doublets which remain unchanged are coloured green (∆δ ≤ 0.04). For CTPR3sw, the mutated residues (NN to RS) are coloured black.

To further explore the thermodynamic response of the C-cap helix in CTPR3 and CTPR3sw to [GuHCl], HSQC spectra were acquired in the presence of denaturant at a level that retains the majority of folded α-helical structure (as determined by CD spectra—1.6 M GuHCl). Under these conditions, it could be expected that the NMR cross-peaks for the majority of the TPR helices would be unchanged. This is true for CTPR3 and, predominantly, for CTPR3sw, where most of the cross-peaks within the first three identical TPR motifs show little change in any specific structure. To compare changes between the spectra at 0 M and 1.6 M, assignable cross-peaks were classified as follows: (i) unchanged, that is, the amide cross-peak at both 0 M and 1.6 M GuHCl resides on the same footprint (chemical shift perturbation ≤ 0.04); (ii) moved such that a chemical shift perturbation could be calculated; and (iii) lost, where “loss” equals a peak that has disappeared or moved into a crowded region of the HSQC and therefore cannot be assigned with certainty (Materials and Methods; Fig. 5). Excitingly, these data show that on addition of a globally destabilising stimulus—GuHCl—CTPR3sw's spectra undergo a specific change that is manifest in the predominant loss of assigned cross-peaks within the C-cap helix (Fig. 5c and d). In contrast, CTPR3 shows little change, specific or otherwise (This is in agreement with a recent single molecule study conducted by the groups of Regan and Haran [47]). Therefore, it can be concluded that CTPR3sw undergoes the reprogrammed unfolding that it was designed to achieve.

## Discussion

### Protein equilibrium unfolding reprogrammed accurately from a statistical mechanics model

In this study, we have shown how the folding energy landscape of designed TPR proteins can be manipulated to reprogramme their multi-state folding. To achieve this, we used a series of helix deletion mutations that permitted a heteropolymer Ising model to characterise and design the system. The data and Ising model analysis highlights the link between inherent structural and thermodynamic asymmetry in our quasi 1-D CTPR/CTPRa proteins. We chose to enhance this asymmetry through the Ising-led introduction of destabilising mutations to create a specific C-terminal helix unfolded intermediate. This gave a CTPR3sw mutant that was experimentally shown to populate a partially unfolded intermediate state whose C-cap helix alone had unfolded. This state closely resembles the CTPR3∆S molecule that has been previously shown to self-associate into fibres when intein ligation groups are incorporated into its termini [40]. As such it constitutes the first step in designing a folding controlled switch-like function into repeat proteins.

### Optimising the population of specific intermediates

**Fig. 6:**
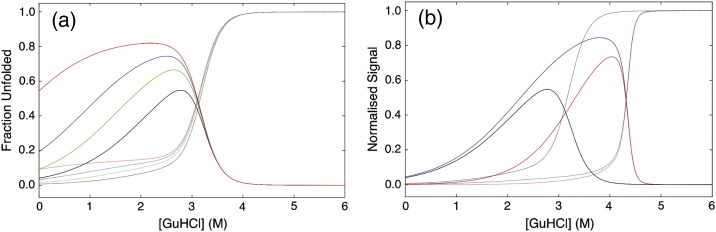
Ising model simulations of CTPR3sw protein compared with (a) CTPR3sw with a C-cap helix that is destabilised by 0.5, 1.0 and 2.0 kcal mol^− 1^ and (b) CTPR10 and CTPR10sw. The simulation of CTPR3sw's denaturation curve is coloured black (dashed line), and the fractional population of CTPR3sw intermediates that only have a denatured C-cap helix is coloured black (solid line). (a) Simulations of CTPR3sw with a C-cap helix that is destabilised by 0.5, 1.0 and 2.0 kcal mol^− 1^ are coloured green, blue and red, respectively. (b) Simulations of CTPR10 and CTPR10sw are coloured red and blue, respectively.

The parameters obtained from global fitting of the deletion mutants to the Ising model can be further used to examine productive routes to a more specific and populated intermediate. Fig. 6 shows simulations of two strategies for improving the reprogrammed folding by (i) additional destabilisation of the C-cap helix relative to the rest of the molecule (CTPR3sw with *G*_*i*_^*S*^ reduced by a further 0.5, 1 and 2 kcal mol^− 1^) or (ii) increasing the total ensemble stability by extending the number of CTPR motifs (CTPR10 and CTPR10sw). Both approaches seek to increase the relative thermodynamic asymmetry between the C-cap and the rest of the molecule. The first strategy does increase the population of C-cap unfolded protein. However, rather than forming a more populated intermediate, the destabilisation causes the C-cap to become natively unfolded. In contrast, the second strategy shows far more promise. It produces intermediates that have higher population, increased efficiency and little natively unfolded C-cap helix. For example, the simulated CTPR10sw protein (10 TPR repeat units plus the C-cap helix from the CTPRa series) produces a C-cap unfolded intermediate population of 88%, of which only 12% is predicted to be natively unfolded. Thus, an effective route to producing the nuanced fulcrum-like behaviour is to increase the number of repeated motifs within a CTPR ensemble.

Given the success of the statistical mechanics Ising model to engineer the desired behaviour in our repeat proteins, the next challenge is to exploit it to produce designed molecular switches by connecting them to trigger a functional output. Previously, we and others have shown that linear repeat proteins can be made to self-associate into higher-order structures and even fibres [40], [48], [49]. Thus, one can envisage a designed repeat protein as a sensor that could dimerise through a specifically triggered local unfolding event in response to environmental stimuli, such as temperature or pH. These switches might produce an amplification of signal *via* self-assembly, making them highly sensitive environmental sensors. Together, the generality of this approach and the applications of these proteins as a self-assembling functional bionanomaterial represent an exciting opportunity.

## Materials and Methods

### Mutagenesis of CTPR/CTPRa constructs

The CTPR/CTPRa constructs lacking the C-cap helix and the switch protein CTPR3sw (NN to RS mutation) were engineered using the quick-change site-directed mutagenesis kit (Stratagene). For the constructs lacking the C-cap helix, a stop codon was inserted so that each construct would be expressed without the helix. CTPRa/CTPR constructs lacking the initially N-cap helix were constructed by PCR, and the new genes were then ligated into the pPROHTbEx expression vector (same vector used for all other constructs).

### Cloning, protein production and purification

All designed CTPR/CTPRa proteins were expressed and purified as previously described [16], [17].

### Structure and sequence of CTPR and CTPRa series of designed TPR proteins

CTPRs were built by arraying multiple copies (*n*) of a 34-aa idealised sequence with a C-terminal single C-cap “solvating” (S) helix. All proteins adopt the distinctive α-helical TPR fold with the unique feature of possessing identical modular structures (Fig. 1b). The CTPR and CTPRa series differ in the loop region between the B-helix of one TPR unit and the A-helix of the next (Fig. 1a). The CTPR series loop encodes P***NN*** and the CTPRa series loop encodes P***RS*** (amino acid positions 33 and 34 in the TPR repeat). Both CTPR and CTPRa series have the same sequence for the C-cap S-helix (AEAKQNLGNAKQKQG). The C-cap helix is based on the sequence of an A-helix in a consensus TPR. However, it differs from an A-helix through the mutation of four solvent exposed large hydrophobic residues (one Trp and three Tyr) to hydrophilic polar residues (Lys and Gln), that is, AEA**WY**NLGNA**YY**KQG to AEA**KQ**NLGNA**KQ**KQG. This was part of the original CTPR design and was implemented to aid solubility [17].

## Biophysical Studies

All biophysical measurements were performed at 10 °C in 50 mM phosphate buffer at pH 7.0, unless otherwise stated.

### Equilibrium chemical denaturation experiments

Far-UV circular dichroism equilibrium unfolding measurements were performed as described previously [9]. For analysis and comparison, the equilibrium curves were normalised using:

(1) $$\text{Normalised}\;\mathrm{CD}\;\text{Signal}=\left\{\text{signal}@222\;\mathrm{nm}-\alpha_{N}\right\}/\left\{\left(\alpha_{D}+\beta_{D}\cdot \left[D\right]\right)-\alpha_{N}\right\}$$

where *α*_D_ and *α*_N_ are the *y*-intercept values of the denatured/native baselines and *β*_D_ is the slope of the denatured baseline. This equation allows the data to retain the slope of the native baseline and be directly globally fit with the heteropolymer Ising model. For all constructs, the slopes of their denatured baselines were not significant.

To assay reproducibility and buffer dependence, the denaturations of the CTPRn protein series were repeated at 10 °C in 50 mM phosphate (pH 7.0) and at 10 °C in 50 mM MOPS (pH 7.0), respectively (Fig. S3). The duplicated denaturations in phosphate buffer gave identical reproducible curves, whereas those in MOPS gave an identical trend as those in phosphate but with midpoints consistently shifted by approximately 0.2 M (S.I. Table S1). This subtle change of midpoint with change in buffer is consistent with other studies showing that differing buffers can give slight differences in chemical denaturation curves [50], [51].

### Equilibrium data analysis

Data were analysed in two specific ways. They were analysed either with a two-state model [9] or with a heteropolymer Ising model [23]. Analysis of the data with a heteropolymer Ising model is described below.

### 1-D Ising model

We initially constructed three derivations of a 1-D heteropolymer Ising model essentially as previously described [23]. Briefly, each model derivation comprises a linear algebraic series of equilibrium constants that account for the intrinsic folding stability (*G*_*i*_) and the interfacial energy (*G*_*i*_ _−_ _1,*i*_) terms of each helix in a nearest-neighbour array that reflects the TPR protein topology. The three models differed on which stability term took a coefficient (*m*) to represent its sensitivity to the external stimulus—in this case, denaturant concentration. In model (i), the denaturant dependence (*m*-value) was associated with the intrinsic stability of each helix (intra-helix—*G_i_*); in model (ii), the denaturant dependence (*m*-value) was associated with the interface energy between folded helices (inter-helix—*G*_*i*_ _−_ _1,*i*_); and in model (iii), a different denaturant dependence (*m*-value) was associated with the intrinsic stability of each helix (intra-helix—*G_i_*) and an interface energy between folded helices (inter-helix—*G*_*i*_ _−_ _1,*i*_). As described below and in detail in the supporting information, the results, figures and discussion shown within this article refer to the Ising model that uses a denaturant-dependent interfacial helix energy.

#### Ising model fitting regime

A numerical solution of each Ising model was determined by globally fitting the normalised chemical denaturation of each CTPR and CTPRa series (normalised using Eq. (1)). A global minimum was ensured by seeding 1000 random searches, each with 1000 trajectories, using the Mathoptimizer module of Mathematica (Wolfram).

#### Ising model parameters

We and others have previously shown that CTPR proteins exhibit fraying of their N- and C-terminal helices under mild equilibrium unfolding conditions. Therefore, we chose to model unique parameters for each of these terminal helices in order that their relative stability could be accounted for and that appropriate sub-populations could be analysed. Internal helices were treated as identical in the model. The asymmetry of CTPR protein folding was modelled *via* unique sets of parameters to represent the N-cap A-helix (*G*_*i*_^A^, *G*_*i* - 1 , *i*_^A^ and *m*^A^), internal I-helices (*G*_*i*_^B^, *G*_*i* − 1 , *i*_^B^ and *m*^B^) and the C-cap S-helix (*G*_*i*_^S^, *G*_*i* − 1 , *i* _^S^ and *m*^S^). The *m* parameters (*m*^A^, *m*^I^ and *m*^S^) gave a denaturant dependence to the intrinsic stabilities [model (i)] and interface energies [model (ii)]. In the case of model (iii), there was an *m* parameter for both *G*_*i*_ and *G*_*i*_ _−_ _1*,i*_ and thus six *m* values in total (*m*^A1^, *m*^A2^ *m*^*I*1^, *m*^I2^, *m*^S1^ and *m*^S2^). The expressions defining the equilibrium constants for each model are as follows: model (i): Eqs. (2), (3), model (ii): Eqs. (4), (5) and model (iii): Eqs. (2), (5).

(2) $$\kappa_{i}=e^{\left[-\left(G_{i}+\left(m\cdot x\right)\right)/\mathrm{RT}\right]}$$

(3) $$\tau_{i-1,i}=e^{\left[-G_{i-1,i}/\mathrm{RT}\right]}$$

(4) $$\kappa_{i}=e^{\left[-G_{i}/RT\right]}$$

(5) $$\tau_{i-1,i}=e^{\left[\left(-G_{i-1,i}+\left(m\cdot x\right)\right)/\mathrm{RT}\right]}$$

where *G*_*i*_ is the free energy of folding for the helix at position *i*, *m* is the denaturant sensitivity at denaturant concentration *x*, *G*_*i* − 1__,*i*_ is the free energy for the interface between helices at positions *i* − 1 and *i*. *R* is the gas constant and *T* is experimental temperature.

The protein partition function for all models, *q*(*n*) is given below (Eq. (6)):

(6) $$q\left(n\right)=\left[0\;1\right]\left[\begin{array}{cc} \kappa_{1}^{A} & v_{1}\\ \kappa_{1}^{A} & v_{1} \end{array}\right]\left[\begin{array}{cc} \kappa_{i}^{I}\cdot \tau_{i-1,i}^{A} & v_{i}\\ \kappa_{i}^{I} & v_{i} \end{array}\right]{\left[\begin{array}{cc} \kappa_{i}^{I}\cdot \tau_{i-1,i}^{I} & v_{i}\\ \kappa_{i}^{I} & v_{i} \end{array}\right]}^{\frac{\left(n-3\right)}{2}}\left[\begin{array}{cc} \kappa_{n}^{S}\cdot \tau_{n-1,n}^{S} & v_{n}\\ \kappa_{n}^{S} & v_{n} \end{array}\right]\left[\begin{array}{c} 1\\ 1 \end{array}\right]$$

The full partition function of the protein with *n* helices is given by *q*(*n*), where all *v* values = 1. This defines the fully folded state. The model allows for fitting of separate parameters (*κ* and *τ*, and thus *G*_*i*_, *G*_*i* - 1 , *i*_ and *m*) to describe the behaviour of the A-, B- and S-helices by globally fitting to data for degenerate CTPR protein compositions. A subpartition function, *q*(*i*), must be calculated by considering only the folded state of each helix in turn, given by iteratively parsing the value *v* = 0 to the term for each helix. The fraction folded, *θ*_F_ is then simply defined as the sum of the subpartition functions divided by the number of terms (helices) multiplied by the full partition function (Eq. (7)):

(7) $$\theta_{F}=\sum_{i=1}^{n}\frac{q\left(i\right)}{n\cdot q\left(n\right)}$$

Normalised chemical denaturations were fitted globally for each CTPR series of proteins of varying numbers of repeating units. Importantly, all models fit the data equally well (as judged by RMSD to the data); produce similar values for the stability of any CTPR ensemble, or part thereof, (∆* G*_0 → 1_^H_2_O^); calculate similar fractional populations of intermediate with an unfolded C-cap helix of CTPR2, CTPR3, CTPRa8 and CTPRa10; and give CTPR3sw simulations that are in extremely good agreement with the experimental equilibrium denaturation data (Tables S2 and S3; Figs. S4 and S5). Moreover, these show that for each CTPR series, the interfacial energies were all stabilising (∆* G*_*i*_ _−_ _1,*i*_^H_2_O^ < 0) and the intrinsic stabilities were not (∆* G*_*i*_^H_2_O^ ≥ 0). From the fitted variables, the stability of any CTPR ensemble or part thereof (∆* G*_0 → 1_^H_2_O^) can be calculated by adding energy terms (Tables 1, S2 and S3). Thus, to obtain a folded CTPR protein, the additive effects of the favourable stabilising interfaces only outweighed the energetically unfavourable intrinsic helix stabilities when ensembles of more than two helices are combined. This is in agreement with previous experimental data analysed with the homozipper Ising model [9], [16], [33].

#### Choice of Ising model

The close agreement of all the models may suggest that the fitting is more based on the combined effects of denaturant dependence, intrinsic and interface energies of each defined helical unit rather than each in isolation. Moreover, when the Ising model that includes separate *m*-values for both intrinsic and interface stabilities was fitted to the CTPRn and CTPRan data, a number of the *m*-values gave values of 0 (Table S2). This would suggest that the data do not include the prerequisite information to support defining them independently. Therefore, it is logical to use the Ising model with fewer parameters. Consequently, the results, figures and discussion shown within this article refer to the Ising model that uses a denaturant-dependent interfacial helix energy. As, arguably, these are mostly energetic whereas the intra-helix term has dominant entropic contributions (reflects the nucleating cost of forming the helix). However, a graphical comparison between fitting of the denaturant-dependent interface Ising model and denaturant-dependent intrinsic Ising model has been added to the supplementary information for completeness (Figs. S4 and S5).

### NMR

^15^N-labelled protein samples (400–800 μM) were prepared in 50 mM phosphate and 150 mM NaCl (pH 6.8) with the addition of 10% D_2_O. Partially denatured protein samples were made up to the desired concentration of denaturant by the addition of 8 M GuHCl. All samples were centrifuged at 14.1 krpm for 10 min to remove particulates prior to pipetting into an NMR tube. Data were collected at 25 °C using a Bruker AV600 600-MHz spectrophotometer. Two-dimensional ^1^H–^15^N HSQC spectra were acquired using Echo/Antiecho-TPPI gradient selection with decoupling during acquisition, using water flip-back pulse with gradients in reverse-inept. Acquisition parameters were as follows: spectral widths, ^15^N, 60 ppm, ^1^H, 14 ppm; 2048 × 128 complex points acquired; acquisition time, 0.12 s; and relaxation delay, 1 s. Spectra were processed using TopSpin 2.1. Published ^1^H–^15^N HSQC spectral assignments were used for CTPR3, as the spectra presented here were acquired under the same pH and buffer conditions used previously [15]. Assignment of the CTPR3sw spectra was achieved by comparing its spectra at 0 M GuHCl with that of CTPR3. These were found to be in good agreement with little perturbation in chemical shifts between the two spectra (Fig. 4d). To further explore the C-cap's structural integrity, both proteins were then incubated with 2 M GuHCl and the HSQC experiments repeated. As predicted from the equilibrium denaturation experiments, CTPR3 undergoes little change between 0 and 2 M GuHCl (Fig. S7). However, 2 M GuHCl changes the CTPR3sw spectra enough to make comparison to its 0 M spectra difficult. Consequently, both CTPR3 and CTPR3sw were repeated with a lower concentration of GuHCl (1.6 M). Here the spectra for CTPR3 mirror that recorded in 2 M (Fig. S7B) and the CTPR3sw spectra were less collapsed than that obtained at 2 M GuHCl. Chemical shift perturbations (∆δ) were calculated with Eq. (8):

(8) $$\mathrm{∆\delta}=\sqrt{\frac{\delta H^{2}+\left(\alpha \cdot \delta N^{2}\right)}{2}}$$

where δ*H* is the difference in the chemical shift for an assigned cross-peak between the two ^1^H spectra, δ*N* is the difference in the chemical shift for an assigned cross-peak between the ^15^N spectra and *α* is a scaling factor that was set at 0.14 [52].

## Figures and Tables

**Table 1:** Values for the global fit of the heteropolymer Ising model to equilibrium chemical denaturation data of CTPRan and CTPRn protein series.

CTPR Series	N-terminal α-helix Cap (A)				Internal α-helices (I)				C-terminal α-helix Cap (S)			
	∆*G*_*i*_^*A*^	∆*G*_*i* − 1,*i*_^*A*^	*m*_A_	a$∆G_{0\to 1}^{H_{2}O}$	∆*G*_*i*_^*I*^	∆*G*_*i* − 1,*i*_^*I*^	*m*_I_	a$∆G_{0\to 1}^{H_{2}O}$	∆*G*_*i*_^*S*^	∆*G*_*i* − 1,*i*_^*S*^	*m*_S_	a$∆G_{0\to 1}^{H_{2}O}$
	$\left(\frac{\mathrm{kcal}}{\mathrm{mol}}\right)$	$\left(\frac{\mathrm{kcal}}{\mathrm{mol}}\right)$	$\left(\frac{\mathrm{kcal}}{\mathrm{molM}}\right)$	$\left(\frac{\mathrm{kcal}}{\mathrm{mol}}\right)$	$\left(\frac{\mathrm{kcal}}{\mathrm{mol}}\right)$	$\left(\frac{\mathrm{kcal}}{\mathrm{mol}}\right)$	$\left(\frac{\mathrm{kcal}}{\mathrm{molM}}\right)$	$\left(\frac{\mathrm{kcal}}{\mathrm{mol}}\right)$	$\left(\frac{\mathrm{kcal}}{\mathrm{mol}}\right)$	$\left(\frac{\mathrm{kcal}}{\mathrm{mol}}\right)$	$\left(\frac{\mathrm{kcal}}{\mathrm{molM}}\right)$	$\left(\frac{\mathrm{kcal}}{\mathrm{mol}}\right)$
CTPRn	5.3	− 9.0	0.9	− 3.7	4.8	− 7.7	0.6	− 2.9	2.95	− 6.3	1.0	− 3.3
CTPRan	2.6	− 6.2	1.3	− 3.6	3.5	− 5.5	0.6	− 1.95	6.0	− 7.7	0.8	− 1.7

The root mean square of residuals from the fit to normalised equilibrium curves for the series of CTPRan varied between 0.02 and 0.05 (equivalent to between < 2 and < 5 % of the data amplitude) and for the series of CTPRn varied between 0.01 and 0.035, equivalent to < 3.5 % of the data amplitude. A single global minimum was ensured by seeding 1000 random searches, each with 1000 trajectories, using the Mathoptimizer module of Mathematica (Wolfram).

a $∆G_{0\to 1}^{H_{2}O}=∆G_{i-1,i}^{\mathrm{helix}\;\mathrm{in}\;H2O}+∆G_{i-1,i}^{\mathrm{helix}\;\mathrm{in}\;H2O}$, that is, $∆G_{0\to 1}^{H_{2}O}$ = the stability gained when a single helix is added to a folded TPR ensemble.

## Appendix A Supplementary data

Supplementary material Image 28

## References

[1] Groves M.R., Barford D. (1999). Topological characteristics of helical repeat proteins. Curr. Opin. Struct. Biol..

[2] Kobe B., Kajava A.V. (2000). When protein folding is simplified to protein coiling: the continuum of solenoid protein structures. Trends Biochem. Sci..

[3] Main E.R., Jackson S.E., Regan L. (2003). The folding and design of repeat proteins: reaching a consensus. Curr. Opin. Struct. Biol..

[4] Varadamsetty G., Tremmel D., Hansen S., Parmeggiani F., Pluckthun A. (2012). Designed Armadillo repeat proteins: library generation, characterization and selection of peptide binders with high specificity. J. Mol. Biol..

[5] Tamaskovic R., Simon M., Stefan N., Schwill M., Pluckthun A. (2012). Designed ankyrin repeat proteins (DARPins) from research to therapy. Methods Enzymol..

[6] Lee S.C., Park K., Han J., Lee J.J., Kim H.J., Hong S. (2012). Design of a binding scaffold based on variable lymphocyte receptors of jawless vertebrates by module engineering. Proc. Natl. Acad. Sci. U. S. A..

[7] Itzhaki L.S., Lowe A.R. (2012). From artificial antibodies to nanosprings: the biophysical properties of repeat proteins. Adv. Exp. Med. Biol..

[8] Urvoas A., Guellouz A., Valerio-Lepiniec M., Graille M., Durand D., Desravines D.C. (2010). Design, production and molecular structure of a new family of artificial alpha-helicoidal repeat proteins (alphaRep) based on thermostable HEAT-like repeats. J. Mol. Biol..

[9] Javadi Y., Main E.R. (2009). Exploring the folding energy landscape of a series of designed consensus tetratricopeptide repeat proteins. Proc. Natl. Acad. Sci. U. S. A..

[10] Tripp K.W., Barrick D. (2008). Rerouting the folding pathway of the Notch ankyrin domain by reshaping the energy landscape. J. Am. Chem. Soc..

[11] Steiner D., Forrer P., Pluckthun A. (2008). Efficient selection of DARPins with sub-nanomolar affinities using SRP phage display. J. Mol. Biol..

[12] Courtemanche N., Barrick D. (2008). The leucine-rich repeat domain of Internalin B folds along a polarized N-terminal pathway. Structure.

[13] Lowe A.R., Itzhaki L.S. (2007). Rational redesign of the folding pathway of a modular protein. Proc. Natl. Acad. Sci. U. S. A..

[14] Main E.R., Lowe A.R., Mochrie S.G., Jackson S.E., Regan L. (2005). A recurring theme in protein engineering: the design, stability and folding of repeat proteins. Curr. Opin. Struct. Biol..

[15] Main E.R., Stott K., Jackson S.E., Regan L. (2005). Local and long-range stability in tandemly arrayed tetratricopeptide repeats. Proc. Natl. Acad. Sci. U. S. A..

[16] Kajander T., Cortajarena A.L., Main E.R., Mochrie S.G., Regan L. (2005). A new folding paradigm for repeat proteins. J. Am. Chem. Soc..

[17] Main E.R., Xiong Y., Cocco M.J., D'Andrea L., Regan L. (2003). Design of stable alpha-helical arrays from an idealized TPR motif. Structure.

[18] Kohl A., Binz H.K., Forrer P., Stumpp M.T., Pluckthun A., Grutter M.G. (2003). Designed to be stable: Crystal structure of a consensus ankyrin repeat protein. Proc. Natl. Acad. Sci. U. S. A..

[19] Forrer P., Stumpp M.T., Binz H.K., Pluckthun A. (2003). A novel strategy to design binding molecules harnessing the modular nature of repeat proteins. FEBS Lett..

[20] Mosavi L.K., Minor D.L., Peng Z.Y. (2002). Consensus-derived structural determinants of the ankyrin repeat motif. Proc. Natl. Acad. Sci. U. S. A..

[21] Cortajarena A.L., Liu T.Y., Hochstrasser M., Regan L. (2010). Designed proteins to modulate cellular networks. ACS Chem. Biol..

[22] Cortajarena A.L., Yi F., Regan L. (2008). Designed TPR modules as novel anticancer agents. ACS Chem. Biol..

[23] Aksel T., Barrick D. (2009). Analysis of repeat-protein folding using nearest-neighbor statistical mechanical models. Methods Enzymol..

[24] Zimm B.H., Bragg J.K. (1959). Theory of the phase transition between helix and random coil in polypeptide chains. J. Chem. Phys..

[25] Munoz V., Thompson P.A., Hofrichter J., Eaton W.A. (1997). Folding dynamics and mechanism of beta-hairpin formation. Nature.

[26] Munoz V., Eaton W.A. (1999). A simple model for calculating the kinetics of protein folding from three-dimensional structures. Proc. Natl. Acad. Sci. U. S. A..

[27] Garcia-Mira M.M., Sadqi M., Fischer N., Sanchez-Ruiz J.M., Munoz V. (2002). Experimental identification of downhill protein folding. Science.

[28] Kubelka J., Henry E.R., Cellmer T., Hofrichter J., Eaton W.A. (2008). Chemical, physical, and theoretical kinetics of an ultrafast folding protein. Proc. Natl. Acad. Sci. U. S. A..

[29] Naganathan A.N., Munoz V. (2014). Thermodynamics of downhill folding: multi-probe analysis of PDD, a protein that folds over a marginal free energy barrier. J. Phys. Chem. B.

[30] Kubelka G.S., Kubelka J. (2014). Site-specific thermodynamic stability and unfolding of a de novo designed protein structural motif mapped by 13C isotopically edited IR spectroscopy. J. Am. Chem. Soc..

[31] Lai J.K., Kubelka G.S., Kubelka J. (2015). Sequence, structure, and cooperativity in folding of elementary protein structural motifs. Proc. Natl. Acad. Sci. U. S. A..

[32] Wetzel S.K., Settanni G., Kenig M., Binz H.K., Pluckthun A. (2008). Folding and unfolding mechanism of highly stable full-consensus ankyrin repeat proteins. J. Mol. Biol..

[33] Phillips J.J., Javadi Y., Millership C., Main E.R. (2012). Modulation of the multistate folding of designed TPR proteins through intrinsic and extrinsic factors. Protein Sci..

[34] Aksel T., Majumdar A., Barrick D. (2011). The contribution of entropy, enthalpy, and hydrophobic desolvation to cooperativity in repeat-protein folding. Structure.

[35] Aksel T., Barrick D. (2014). Direct observation of parallel folding pathways revealed using a symmetric repeat protein system. Biophys. J..

[36] Werbeck N.D., Itzhaki L.S. (2007). Probing a moving target with a plastic unfolding intermediate of an ankyrin-repeat protein. Proc. Natl. Acad. Sci. U. S. A..

[37] Cortajarena A.L., Mochrie S.G., Regan L. (2008). Mapping the energy landscape of repeat proteins using NMR-detected hydrogen exchange. J. Mol. Biol..

[38] Cortajarena A.L., Regan L. (2011). Calorimetric study of a series of designed repeat proteins: modular structure and modular folding. Protein Sci..

[39] Krachler A.M., Sharma A., Kleanthous C. (2010). Self-association of TPR domains: lessons learned from a designed, consensus-based TPR oligomer. Proteins.

[40] Phillips J.J., Millership C., Main E.R. (2012). Fibrous nanostructures from the self-assembly of designed repeat protein modules. Angew. Chem. Int. Ed. Engl..

[41] Gasteiger E., Hoogland C., Gattiker A., Duvaud S., Wilkins M.R., Appel R.D., JM Walker (2005). Protein Identification and Analysis Tools on the ExPASy Server. The Proteomics Protocols Handbook.

[42] Cortajarena A.L., Wang J., Regan L. (2010). Crystal structure of a designed tetratricopeptide repeat module in complex with its peptide ligand. FEBS J..

[43] Naganathan A.N., Munoz V. (2008). Determining denaturation midpoints in multiprobe equilibrium protein folding experiments. Biochemistry.

[44] Sadqi M., Fushman D., Munoz V. (2006). Atom-by-atom analysis of global downhill protein folding. Nature.

[45] Naganathan A.N., Perez-Jimenez R., Sanchez-Ruiz J.M., Munoz V. (2005). Robustness of downhill folding: guidelines for the analysis of equilibrium folding experiments on small proteins. Biochemistry.

[46] Oliva F.Y., Munoz V. (2004). A simple thermodynamic test to discriminate between two-state and downhill folding. J. Am. Chem. Soc..

[47] Cohen S.S., Riven I., Cortajarena A.L., De Rosa L., D'Andrea L.D., Regan L. (2015). Probing the molecular origin of native-state flexibility in repeat proteins. J. Am. Chem. Soc..

[48] Watson R.P., Christen M.T., Ewald C., Bumbak F., Reichen C., Mihajlovic M. (2014). Spontaneous self-assembly of engineered armadillo repeat protein fragments into a folded structure. Structure.

[49] Voet A.R., Noguchi H., Addy C., Simoncini D., Terada D., Unzai S. (2014). Computational design of a self-assembling symmetrical beta-propeller protein. Proc. Natl. Acad. Sci. U. S. A..

[50] Campos L.A., Munoz V. (2013). Reply to Huang et al.: Slow proton exchange can duplicate the number of species observed in single-molecule experiments of protein folding. Proc. Natl. Acad. Sci. U. S. A..

[51] Huang F., Johnson C.M., Petrovich M., Fersht A.R. (2013). Don't waste good methods on bad buffers and ambiguous data. Proc. Natl. Acad. Sci. U. S. A..

[52] Williamson M.P. (2013). Using chemical shift perturbation to characterise ligand binding. Prog. Nucl. Magn. Reson. Spectrosc..

