# Supplementary material for: Ising Model Reprogramming of a Repeat Protein's Equilibrium Unfolding Pathway
Source: J Mol Biol. 2016 May 8;428(9Part A):1804–17. doi: 10.1016/j.jmb.2016.02.022 (PMC4871810; doi:10.1016/j.jmb.2016.02.022)
Supplement: Supplementary file 1 — Supplementary material [file mmc1.pdf]

Supplementary Information

SI Table 1: Comparison of the parameters obtained from fitting the GuHCl induced unfolding of CTPR proteins using a two state model at 10 ^{O}C.

**Construct**<br>CTPRn Series	**50 mM Phosphate pH 7 Dataset 1**		
	$[D]_{50\%}$ (M)	*m*-value (kcalmol^{-1}M^{-1})	ΔG_{D-N}^{H2O} (kcalmol^{-1})
CTPR2ΔA	1.94 ± 0.01	2.08 ± 0.05	4.0 ± 0.1
CTPR2ΔS	2.21 ± 0.01	2.10 ± 0.03	4.6 ± 0.1
CTPR2	2.57 ± 0.01	2.43 ± 0.06	6.2 ± 0.2
CTPR3ΔA	2.97 ± 0.01	2.8 ± 0.1	8.4 ± 0.3
CTPR3ΔS	3.17 ± 0.01	2.6 ± 0.08	8.4 ± 0.3
CTPR3	3.36 ± 0.01	2.9 ± 0.1	9.7 ± 0.4
	**50 mM Phosphate pH 7 Dataset 2**		
CTPRn Series	$[D]_{50\%}$ (M)	*m*-value (kcalmol^{-1}M^{-1})	ΔG_{D-N}^{H2O} (kcalmol^{-1})
CTPR2ΔA	1.99 ± 0.02	2.08 ± 0.08	4.2 ± 0.2
CTPR2ΔS	2.24 ± 0.02	2.2 ± 0.15	4.9 ± 0.3
CTPR2	2.62 ± 0.01	2.55 ± 0.06	6.7 ± 0.2
CTPR3ΔA	2.99 ± 0.01	2.5 ± 0.1	7.5 ± 0.3
CTPR3ΔS	3.17 ± 0.01	2.7 ± 0.13	8.6 ± 0.4
CTPR3	3.29 ± 0.01	3.6 ± 0.13	11.9 ± 0.4
	**50 mM MOPS pH 7**		
CTPRn Series	$[D]_{50\%}$ (M)	*m*-value (kcalmol^{-1}M^{-1})	ΔG_{D-N}^{H2O} (kcalmol^{-1})
CTPR2ΔA	1.81 ± 0.01	2.09 ± 0.05	3.8 ± 0.1
CTPR2ΔS	2.04 ± 0.01	2.01 ± 0.05	4.1 ± 0.1
CTPR2	2.42 ± 0.01	2.4 ± 0.07	5.9 ± 0.2
CTPR3ΔA	2.67 ± 0.01	2.8 ± 0.07	7.4 ± 0.2
CTPR3ΔS	3.07 ± 0.01	3.0 ± 0.11	9.1 ± 0.3
CTPR3	3.14 ± 0.01	3.0 ± 0.17	9.4 ± 0.5
	**50 mM Phosphate pH 7**		
CTPRa Series	$[D]_{50\%}$ (M)	*m*-value (kcalmol^{-1}M^{-1})	ΔG_{D-N}^{H2O} (kcalmol^{-1})
CTPRa2ΔA	1.01 ± 0.04	1.74 ± 0.05	1.8 ± 0.1
CTPRa2ΔS	1.63 ± 0.02	1.87 ± 0.07	3.0 ± 0.1
[a]CTPRa2	1.71 ± 0.04	2.1 ± 0.2	3.5 ± 0.3
CTPRa3ΔA	2.18 ± 0.01	2.34 ± 0.08	5.1 ± 0.2
CTPRa3ΔS	2.31 ± 0.01	2.30 ± 0.09	5.3 ± 0.3
[a]CTPRa3	2.39 ± 0.02	2.4 ± 0.12	5.8 ± 0.3

$[D]_{50\%}$ and *m*-values were calculated by fitting data to a two state equation (Main et al., 1998). $\Delta G_{\mathrm{D-N}}^{\mathrm{H_2O}}$ were calculated using individual *m*-values. The errors reported are from the fitting of the experimental data and propagation of the fitting errors there after. [a]Previously reported in Main *et al*. (Javadi and Main, 2009).

SI Table 2: Comparison of the parameters obtained from fitting the GuHCl induced unfolding of CTPR/CTPRan proteins to 3 formulations of the Heteropolymer Ising model (differ in whether (i) the intrinsic helix energy, (ii) interfacial interaction energy or (iii) both intrinsic & interface energies have denaturant dependence) in 50 mM Phosphate pH 7 at 10 ^{O}C.

Ising Model using a denaturant dependent intrinsic helix stability ($\boldsymbol{m}$ & $\boldsymbol{\Delta G_i}$)

CTPR	N-terminal α-helix Cap (A)				Internal α-helices (I)				C-terminal α-helix Cap (S)			
Series	ΔG_i^A $\left(\frac{\text{kcal}}{\text{mol}}\right)$	m_{A} (i) $\left(\frac{\text{kcal}}{\text{molM}}\right)$	$\Delta G_{i-1,i}^A$ $\left(\frac{\text{kcal}}{\text{mol}}\right)$	[a]$\Delta G_{0\to1}^{H_2O}$ $\left(\frac{\text{kcal}}{\text{mol}}\right)$	ΔG_i^I $\left(\frac{\text{kcal}}{\text{mol}}\right)$	m_{I} (i) $\left(\frac{\text{kcal}}{\text{molM}}\right)$	$\Delta G_{i-1,i}^I$ $\left(\frac{\text{kcal}}{\text{mol}}\right)$	[a]$\Delta G_{0\to1}^{H_2O}$ $\left(\frac{\text{kcal}}{\text{mol}}\right)$	ΔG_i^S $\left(\frac{\text{kcal}}{\text{mol}}\right)$	m_{S} (i) $\left(\frac{\text{kcal}}{\text{molM}}\right)$	$\Delta G_{i-1,i}^S$ $\left(\frac{\text{kcal}}{\text{mol}}\right)$	[a]$\Delta G_{0\to1}^{H_2O}$ $\left(\frac{\text{kcal}}{\text{mol}}\right)$
CTPRn	5.7	0.6	-8.8	-3.1	3.5	0.5	-6.1	-2.6	0.0	0.8	-2.9	-2.9
CTPRan	5.6	0.9	-8.2	-2.6	2.3	0.5	-4.0	-1.7	0.4	0.7	-2.0	-1.6

Ising Model using a denaturant dependent helix interface stability ($\boldsymbol{m}$ & $\boldsymbol{\Delta G_{i-1,i}}$)

CTPR	N-terminal α-helix Cap (A)				Internal α-helices (I)				C-terminal α-helix Cap (S)			
Series	ΔG_i^A $\left(\frac{\text{kcal}}{\text{mol}}\right)$	$\Delta G_{i-1,i}^A$ $\left(\frac{\text{kcal}}{\text{mol}}\right)$	m_{A} (i-1,i) $\left(\frac{\text{kcal}}{\text{molM}}\right)$	[a]$\Delta G_{0\to1}^{H_2O}$ $\left(\frac{\text{kcal}}{\text{mol}}\right)$	ΔG_i^I $\left(\frac{\text{kcal}}{\text{mol}}\right)$	$\Delta G_{i-1,i}^I$ $\left(\frac{\text{kcal}}{\text{mol}}\right)$	m_{I} (i-1,i) $\left(\frac{\text{kcal}}{\text{molM}}\right)$	[a]$\Delta G_{0\to1}^{H_2O}$ $\left(\frac{\text{kcal}}{\text{mol}}\right)$	ΔG_i^S $\left(\frac{\text{kcal}}{\text{mol}}\right)$	$\Delta G_{i-1,i}^S$ $\left(\frac{\text{kcal}}{\text{mol}}\right)$	m_{S} (i-1,i) $\left(\frac{\text{kcal}}{\text{molM}}\right)$	[a]$\Delta G_{0\to1}^{H_2O}$ $\left(\frac{\text{kcal}}{\text{mol}}\right)$
CTPRn	5.3	-9.0	0.9	-3.7	4.8	-7.7	0.6	-2.9	2.95	-6.3	1.0	-3.3
CTPRan	2.6	-6.2	1.3	-3.6	3.5	-5.5	0.6	-1.95	6.0	-7.7	0.8	-1.7

Ising Model using denaturant dependence for both intrinsic & interface helix stabilities

CTPR	N-terminal α-helix Cap (A)					Internal α-helices (I)					C-terminal α-helix Cap (S)				
Series	ΔG_i^A $\left(\frac{\text{kcal}}{\text{mol}}\right)$	m_{A} (i) $\left(\frac{\text{kcal}}{\text{molM}}\right)$	$\Delta G_{i-1,i}^A$ $\left(\frac{\text{kcal}}{\text{mol}}\right)$	m_{A} (i-1,i) $\left(\frac{\text{kcal}}{\text{molM}}\right)$	[a]$\Delta G_{0\to1}^{H_2O}$ $\left(\frac{\text{kcal}}{\text{mol}}\right)$	ΔG_i^I $\left(\frac{\text{kcal}}{\text{mol}}\right)$	m_{I} (i) $\left(\frac{\text{kcal}}{\text{molM}}\right)$	$\Delta G_{i-1,i}^I$ $\left(\frac{\text{kcal}}{\text{mol}}\right)$	m_{I} (i-1,i) $\left(\frac{\text{kcal}}{\text{molM}}\right)$	[a]$\Delta G_{0\to1}^{H_2O}$ $\left(\frac{\text{kcal}}{\text{mol}}\right)$	ΔG_i^S $\left(\frac{\text{kcal}}{\text{mol}}\right)$	m_{S} (i) $\left(\frac{\text{kcal}}{\text{molM}}\right)$	$\Delta G_{i-1,i}^S$ $\left(\frac{\text{kcal}}{\text{mol}}\right)$	m_{S} (i-1,i) $\left(\frac{\text{kcal}}{\text{molM}}\right)$	[a]$\Delta G_{0\to1}^{H_2O}$ $\left(\frac{\text{kcal}}{\text{mol}}\right)$
CTPRn	4.1	0.4	-7.5	0.4	-3.3	3.5	0.5	-6.0	0.0	-2.5	1.0	0.4	-4.0	0.45	-2.9
CTPRan	2.2	0.2	-5.7	1.1	-3.5	3.5	0.0	-5.5	0.6	-2.0	5.3	0.0	-7.0	0.8	-1.7

[a]$\Delta G_{0\to1}^{H_2O} = \Delta G_{i-1,i}^{helix\ in\ H2O} + \Delta G_{i-1,i}^{helix\ in\ H2O}$ i.e. The stability gained when a single helix is added to a folded TPR ensemble. The root mean square of residuals from the fit to normalised equilibrium curves for the series of CTPRan varied between 0.02 and 0.05 (equivalent to between < 2 and < 5 % of the data amplitude) and for the series of CTPRn varied between 0.01 and 0.035, equivalent to < 3.5 % of the data amplitude. A single global minimum was ensured by seeding 1000 random searches, each with 1000 trajectories, using the Mathoptimizer module of Mathematica (Wolfram

SI Table 3: Comparison of the stability of any CTPR ensemble, or part thereof, ($\Delta G_{0\to j}^{H20}$) obtained from fitting the GuHCl induced unfolding of CTPR/CTPRan proteins with 3 formulations of the Heteropolymer Ising model (differ in whether (i) the intrinsic helix energy, (ii) interfacial interaction energy or (iii) both intrinsic & interface energies have denaturant dependence) in 50 mM Phosphate pH 7 at 10 ^{O}C.

**CTPRn Series**	[D] dependent intrinsic helix stability (m & ΔG_i)	[D] dependent interface stability (m & $\Delta G_{i-1,i}^{I}$)	[D] dependent intrinsic & interface stability (m & ΔG_i , m & $\Delta G_{i-1,i}^{I}$)
	$^{a}\Delta \boldsymbol{G_{0\to j}^{H20}}$ **(kcalmol^{-1})**	$^{a}\Delta \boldsymbol{G_{0\to j}^{H20}}$ **(kcalmol^{-1})**	$^{a}\Delta \boldsymbol{G_{0\to j}^{H20}}$ **(kcalmol^{-1})**
A Helix (onto a folded ensemble)	-3.1	-3.7	-3.3
I Helix (onto a folded ensemble)	-2.6	-2.9	-2.5
S Helix (onto a folded ensemble)	-2.9	-3.3	-2.9
CTPR2ΔA	-4.6	-4.3	-4.5
CTPR2ΔS	-4.8	-4.7	-4.9
CTPR2	-7.7	-8.0	-7.8
CTPR3ΔA	-9.8	-10.2	-9.5
CTPR3ΔS	-10.0	-10.5	-10.0
CTPR3	-12.9	-13.8	-12.9
**CTPRan Series**			
A Helix (onto a folded ensemble)	-2.6	-3.6	-3.5
I Helix (onto a folded ensemble)	-1.7	-2.0	-2.0
S Helix (onto a folded ensemble)	-1.6	-1.7	-1.7
CTPRa2ΔS	-3.8	-3.9	-3.9
CTPRa2	-5.4	-5.6	-5.6
CTPRa3ΔA	-6.2	-6.0	-6.0
CTPRa3ΔS	-7.2	-7.8	-7.8
CTPRa3	-8.8	-9.5	-9.5
CTPRa4	-12.3	-13.4	-13.4
CTPRa5	-15.7	-17.3	-17.3
CTPRa6	-19.2	-21.2	-21.2
CTPRa8	-26.1	-29.0	-29.0
CTPRa10	-33.0	-32.9	-32.9

$^{a}\Delta G_{0\to j}^{H20} = \left[\Delta G_i^{A} + \Delta G_{i-1,i}^{A}\right] + \left[n\Delta G_i^{I} + (n-1)\Delta G_{i-1,i}^{I}\right] + \left[\Delta G_i^{S} + \Delta G_{i-1,i}^{S}\right]$

Supplementary Figure 1:

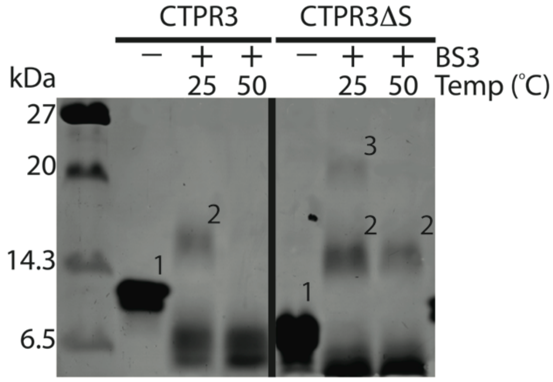

S.I. Figure 1: SDS PAGE gel of CTPR3 and CTPR3ΔS crosslinking studies. 20:1 molar ratio of Bis[sulfosuccinimidyl] suberate (BS3; Pierce) crosslinker:protein were incubated at either 25 or 50 ˚C for 30 mins in 50 mM phosphate buffer pH 7.0, prior to quenching with Tris pH 7.0. Monomeric species (1) are observed in the absence of crosslinker, but following incubation with BS3 dimeric (2) and trimeric (3) species were detected. The full-length CTPR3 dimerised in the presence of crosslinker. The equivalent protein lacking the C-terminal S-helix, CTPR3ΔS, yielded both dimer and trimer species following identical incubation with crosslinker. At higher temperatures less crosslinking is observed as the crosslinker is more quickly hydrolysed.

Supplementary Figure 2:

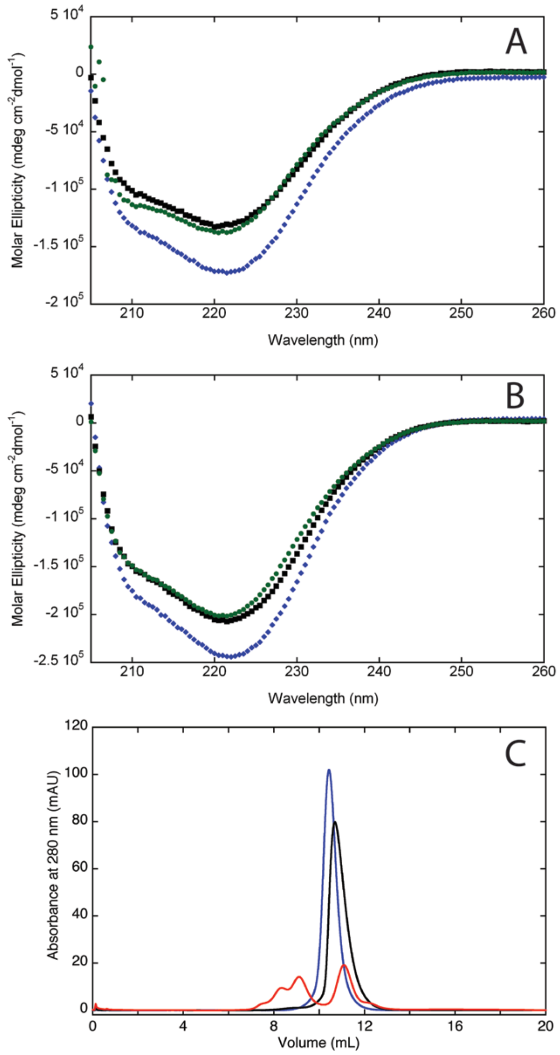

S.I Figure 2: Examples of the secondary structure and molecular size of the ΔA and ΔS CTPRn mutants (A) Far UV wavelength scans of CTPR2 (blue), CTPR2ΔA (green) and CTPR2ΔS (black). (B) Far UV wavelength scans of CTPR3 (blue), CTPR3ΔA (green) and CTPR3ΔS (black). All C.D. samples were in 50 mM phosphate pH 7 and were performed in a thermostated cuvette holder at 10 ^{o}C. The scan showed a single negative peak at 222 nm, indicating that the protein is highly α-helical. (C) Size Exclusion Chromatography (SEC) of 100 µL of 100 µM protein injected onto a Superdex G75 10/30 analytical column. CTPR3 (blue), CTPR3ΔS (black) and four protein standards (red – peaks for Albumin – 67kDa, Ovalbumin – 43kDa, Chymotrypsinogen A – 25kDa and Ribonclease A – 16.4 kDa). As can be seen both proteins elute as monomeric peaks.

Supplementary Figure 3:

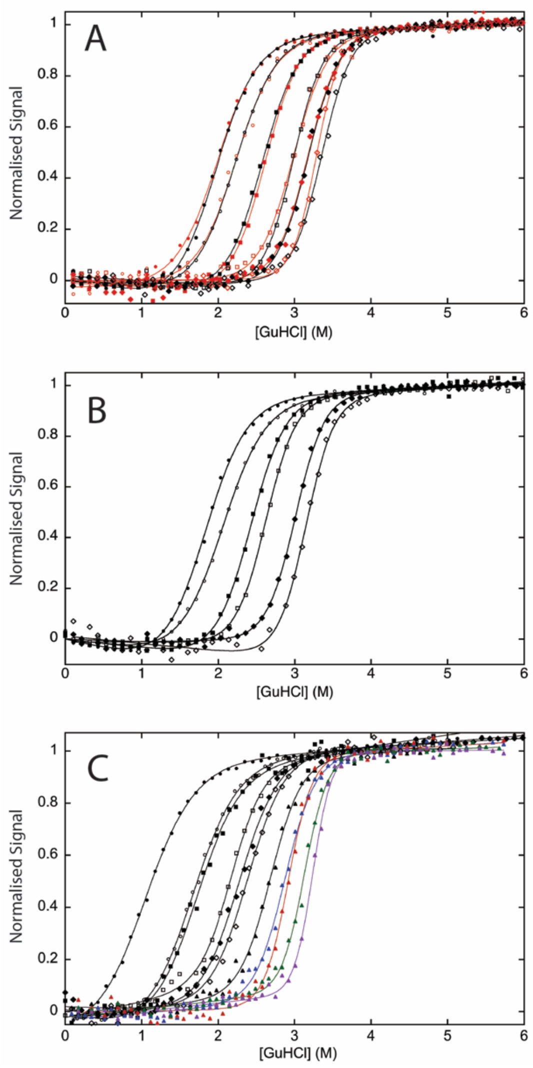

S.I Figure 3: GuHCl-induced equilibrium unfolding experiments of the CTPRn (A & B) & CTPRa (C) proteins at 10 °C. For comparison all equilibrium curves were normalised using: Normalised Signal = (signal @ 222nm − α_N)/ (α_D – α_N), where α_D and α_N are the y-intercept values of the denatured and native baselines. This equation allows for the data to retain the slope of the folded and unfolded baselines. Solid lines correspond to the best fit of a two-state folding model.

(A) Fraction unfolded versus [GuHCl] for the CTPRn series performed in 50 mM phosphate pH 7. (B) Fraction unfolded versus [GuHCl] for the CTPRn series in 50 mM MOPS pH 7. (C) Fraction unfolded versus [GuHCl] for the CTPRan series performed in 50 mM phosphate pH 7:

In (A), (B) and (C): CTPR2ΔA (filled circles – red & black), CTPR2ΔS (open circles – red & black), CTPR2 (filled squares – red & black), CTPR3ΔA (open squares – red & black), CTPR3ΔS (filled diamonds – red & black), CTPR3 (open diamonds – red & black), CTPRa2ΔA (filled circles), CTPRa2ΔS (open circles), CTPRa3ΔA (open squares) and CTPRa3ΔS (filled diamonds). The following data were obtained from published data in Main *et al*. (Javadi and Main, 2009): CTPRa2 (filled squares), CTPRa3 (open diamonds), CTPRa4 (black triangles), CTPRa5 (blue triangles), CTPRa6 (red triangles), CTPRa8 (green triangles) and CTPRa10 (purple triangles).

Supplementary Figure 4:

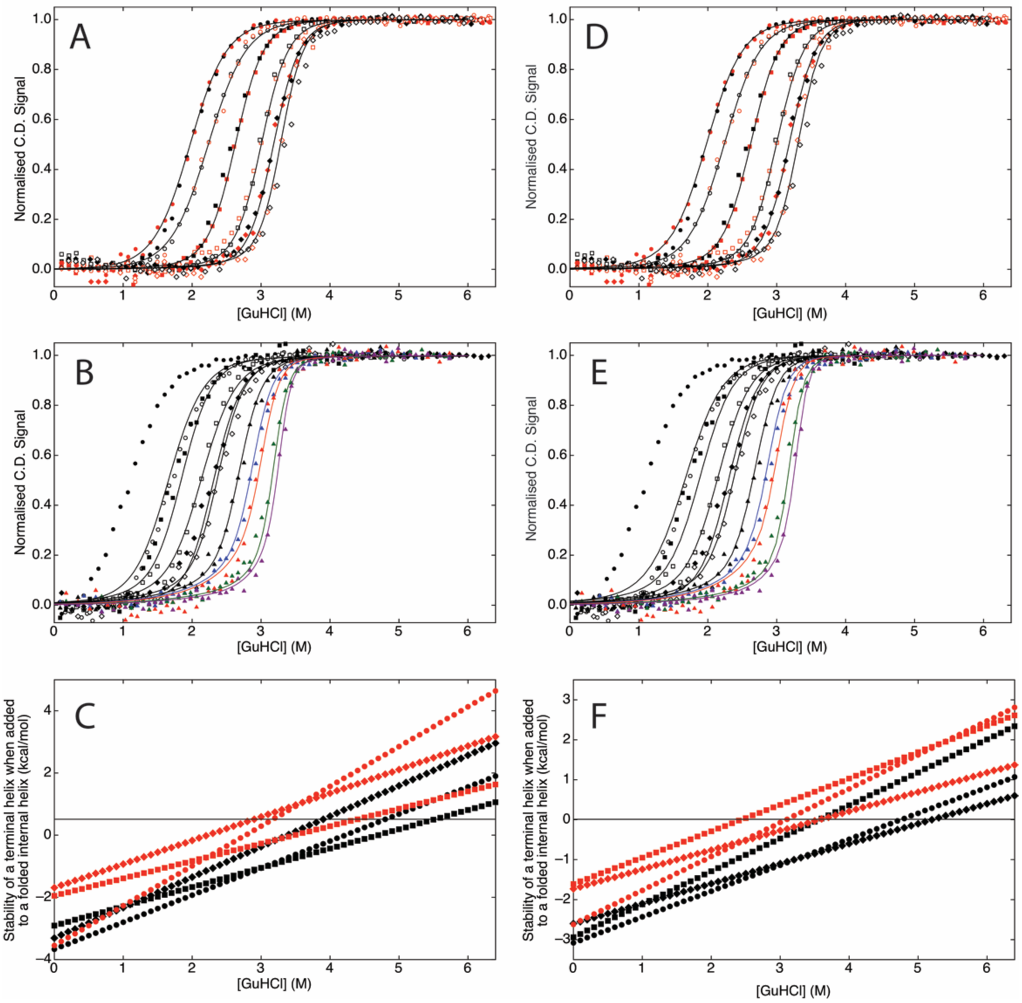

S.I Figure 4:

(A & E) GuHCl-induced equilibrium unfolding experiments of the CTPRn (A & D) & CTPRa (B & E) proteins in 50 mM phosphate pH 7 at 10 °C. Plots A, B and C show equilibrium unfolding curves fit to the heteropolymer Ising Model that has a denaturant dependent helix interface stability (m & $\Delta G_{i-1,i}$). Whereas plots D, E and F show equilibrium unfolding curves fit to the heteropolymer Ising Model that has a denaturant dependent intrinsic helix stability (m & ΔG_i). In (A - D): CTPR2ΔA (●,●), CTPR2ΔS (○,○), CTPR2 (■, ■), CTPR3ΔA (□,□), CTPR3ΔS (◆,◆) and CTPR3 (◇,◇). CTPRa2ΔA (●), CTPRa2ΔS (○), CTPRa3ΔA (□) and CTPRa3ΔS (◆). The following data were obtained from published data in Main et al. [9]: CTPRa2 (■), CTPRa3 (◇), CTPRa4 (▲), CTPRa5 (▲), CTPRa6 (▲), CTPRa8 (▲) and CTPRa10 (▲).

(C & F) Stabilities of terminal helices added to a folded CTPR ensemble as a function of GuHCl. Plot C shows values obtained from fitting the data to the heteropolymer Ising Model that has a denaturant dependent helix interface stability (m & $\Delta G_{i-1,i}$). Whereas F show shows values obtained from fitting the data to the heteropolymer Ising Model that has a denaturant dependent intrinsic helix stability (m & ΔG_i). An N-cap helix (■, ■), I helix (◆,◆) and a C-cap Helix (●,●). The CTPRn & CTPRa series are black and red, respectively.

Supplementary Figure 5:

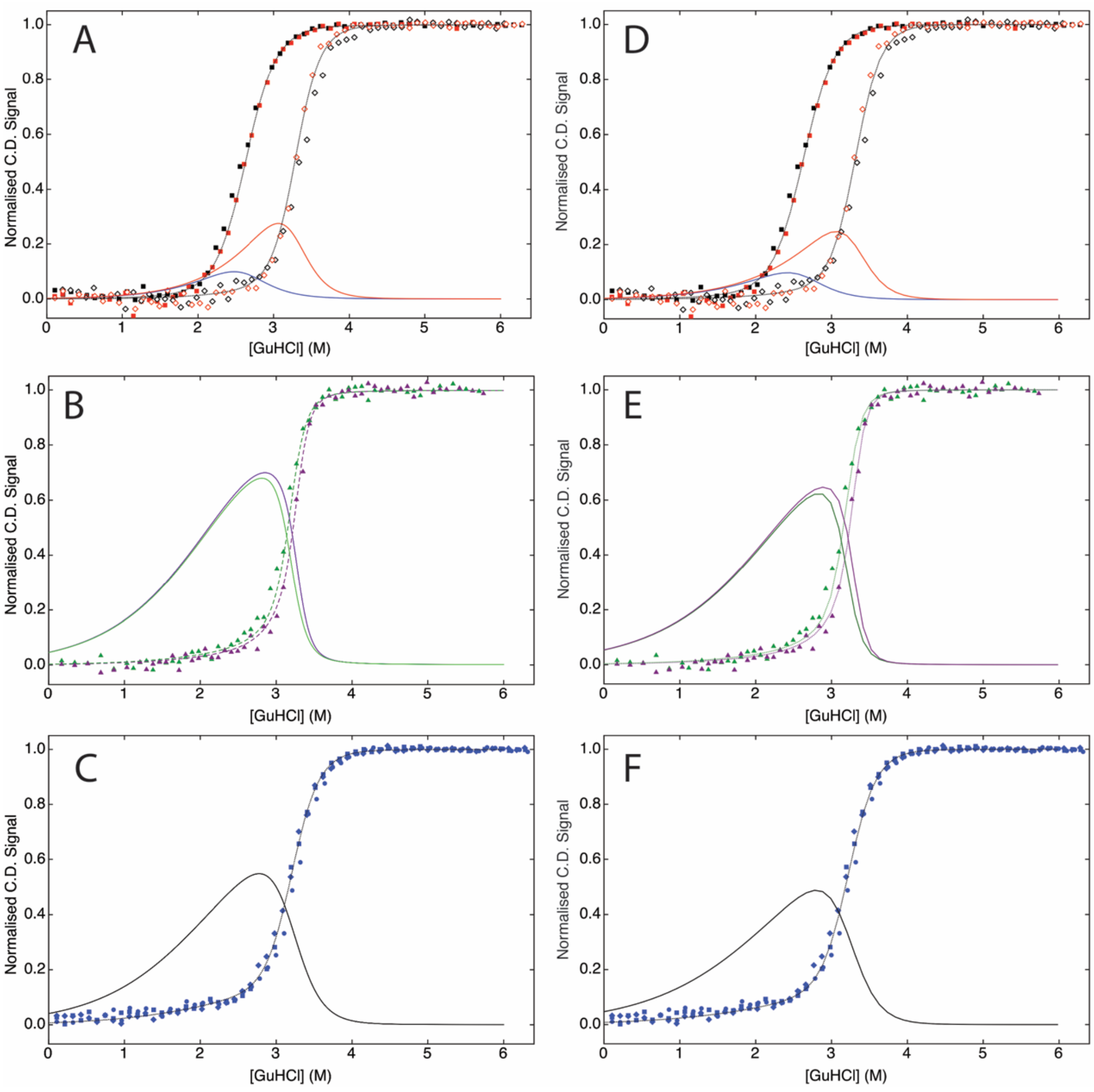

S.I Figure 5:

(A, B, D & E) Ising model obtained fractional population of intermediates that only have a denatured C-cap helix for (A & D) CTPR2 (solid blue line) and CTPR3 (solid red line) and (B & E) CTPRa8 (solid green line) and CTPRa10 (solid purple line).

(C & F) Comparison of the GuHCl denaturations of CTPR3sw (●, ■ and ◆) with the simulated CTPR3sw denaturation curve obtained from the Ising model (black small dashed line) and the fractional population of CTPR3sw intermediates that only have an denatured C-cap helix [solid line].

Plots A- C correspond to values obtained from the heteropolymer Ising Model that has a denaturant dependent helix interface stability (m & $\Delta G_{i-1,i}$). Plots D - F correspond to values obtaind from the heteropolymer Ising Model that has a denaturant dependent intrinsic helix stability (m & ΔG_i). The corresponding CTPR protein chemical denaturations with Ising fit (black dashed line) are shown for comparison.

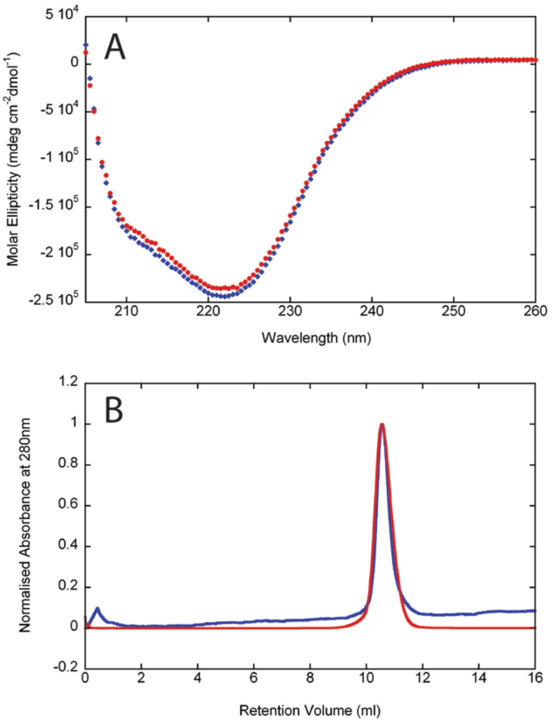

S.I Figure 6:

(A) Far UV wavelength scans of CTPR3 (blue), CTPR3sw (red). All C.D. samples were in 50 mM phosphate pH 7 and were held in a thermostated cuvette holder at 10 °C.

(B) Size Exclusion Chromatography (SEC) of 100 µL of 100 µM protein injected onto a Superdex G75 10/30 analytical column. CTPR3 (blue) and CTPR3sw (red).

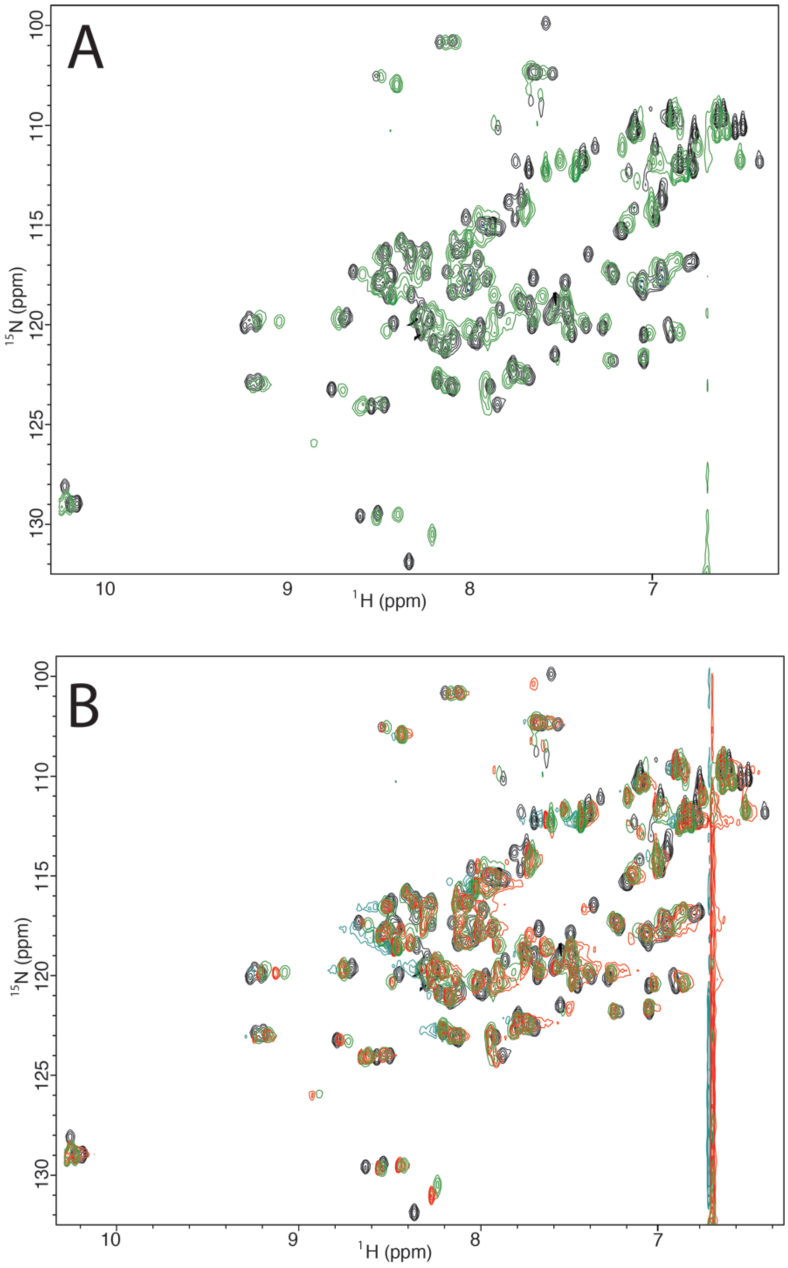

S.I Figure 7:

(A) Overlaid HSQC NMR spectra of CTPR3 in 0 M GuHCl (black) and 2 M GuHCl (green)

(B) Overlaid HSQC NMR spectra of CTPR3 in 0 M GuHCl (black), 1.6 M GuHCl (red) and 2 M GuHCl (green). Sample conditions were 50 mM phosphate pH 6.8, 150 mM NaCl. Data was recorded at room temperature.

REFERENCES

Javadi, Y., and Main, E.R. (2009). Exploring the folding energy landscape of a series of designed consensus tetratricopeptide repeat proteins. P Natl Acad Sci Usa *106*, 17383-17388.

Main, E.R., Fulton, K.F., and Jackson, S.E. (1998). Context-dependent nature of destabilizing mutations on the stability of FKBP12. Biochemistry *37*, 6145-6153.
